# ROS1 Fusion Mediates Immunogenicity by Upregulation of PD-L1 After the Activation of ROS1–SHP2 Signaling Pathway in Non-Small Cell Lung Cancer

**DOI:** 10.3389/fimmu.2020.527750

**Published:** 2020-11-25

**Authors:** Liangliang Cai, Jianchun Duan, Li Qian, Zhijie Wang, Shuhang Wang, Sini Li, Chao Wang, Jie Zhao, Xue Zhang, Hua Bai, Jie Wang

**Affiliations:** ^1^ Institute of Translational Medicine, Medical College, Yangzhou University, Yangzhou, China; ^2^ Jiangsu Key Laboratory of Experimental & Translational Non-coding RNA Research, Yangzhou, China; ^3^ National Cancer Center/National Clinical Research Center for Cancer/Cancer Hospital, Chinese Academy of Medical Sciences and Peking Union Medical College, Beijing, China

**Keywords:** PD-L1, ROS1 fusion, ROS1-G2032R mutation, non-small cell lung cancer, molecular targeted therapy, immunology checkpoint inhibitor therapy

## Abstract

The drug resistance of first-line crizotinib therapy for ROS proto-oncogene 1, receptor tyrosine kinase (ROS1) fusion non-small cell lung cancer (NSCLC) is inevitable. Whether the administration of immune checkpoint inhibitor (ICI) therapy is suitable for ROS 1 fusion NSCLCs or after the development of crizotinib resistance is still unknown. In this study, five different crizotinib resistant concentration cell lines (HCC78CR1-5) from primary sensitive HCC78 cells were cultured. Ba/F3 cells expressing crizotinib sensitive ROS1 fusion and crizotinib resistant ROS1-G2032R mutation were used to explore the relationship between ROS1 fusion, ROS1-G2032R mutation and programmed death-ligand 1 (PD-L1) expression and the clinical potential of anti-PD-L1 ICI therapy. The signaling pathway net was compared between HCC78 and HCC78CR1-5 cells using RNA sequencing. Anti- PD-L1 ICI therapy was performed on mouse xenograft models with Ba/F3 ROS1 fusion or ROS1-G2032R mutation. HCC78CR1-5 showed more immunogenicity than HCC78 in immune-related pathways. The PD-L1 expression level was remarkably higher in HCC78CR1-5 with ROS1 fusion upregulation than HCC78 primary cell. Furthermore, the expression of PD-L1 was down-regulated by RNA interference with ROS1 siRNAs and up-regulated lower in Ba/F3 ROS1-G2032R resistant mutation than ROS1 fusion. Western blotting analysis showed the ROS1–SHP2 signaling pathway activation in HCC78CR1-5 cells, Ba/F3 ROS1 fusion and ROS1-G2032R resistant mutation. Mouse xenograft models with Ba/F3 ROS1 fusion showed more CD3+PD-1+ T cells both in blood and tissue, and more sensitivity than the cells with Ba/F3 ROS1-G2032R resistant mutation after anti-PD-L1 therapy. Our findings indicate that PD-L1 upregulation depends on ROS1 fusion more than ROS1-G2032R mutation. We share our insights of NSCLCs treatment management into the use of anti-PD-L1 ICI therapy in ROS1 fusion and not in ROS1-G2032R resistant mutation.

## Introduction

At present, molecular targeted therapy is an important strategy for the treatment of non-small cell lung cancer (NSCLC) (1, 2). Driver genes play central roles in tumorigenesis and tumor cell survival and proliferation. Chromosomal rearrangement involving the Solute Carrier Family 34 Member 2 (SLC34A2) and ROS proto-oncogene 1, receptor tyrosine kinase (ROS1) genes defines a distinct molecular subset of NSCLCs which possess over 14% of all ROS1 fusion type, with the resulting fusion gene manifesting pronounced transforming activity. Crizotinib is the first-line treatment for ROS1 fusion rearrangement positive NSCLCs.

ROS1 fusion NSCLCs are sensitive to crizotinib, but their development of drug resistance is inevitable. As a new drug class, immunology checkpoint inhibitor (ICI) therapies, such as those involving anti-CTLA4 (3), anti-programmed cell death protein 1 (PD-1) (4) and anti-programmed death-ligand 1 (PD-L1) (5), show promise in the clinical treatment of several cancer types, especially melanoma and lung cancer (6), and they cause cancer to become a chronic disease by activating tumor immune cells in the tumor microenvironment (TME). In anti-PD-L1 therapy, the expression level of PD-L1 on tumor cells especially on tumor cells’ surface, is a key biomarker that correlates with the likelihood of an effective clinical response (7). PD-L1 expression in NSCLCs is up-regulated by epidermal growth factor receptor (EGFR) mutation, implicating oncogenic drivers in the regulation of the expression of PD-L1, which is an important immunosuppressive molecule (8, 9). NSCLCs exhibiting upregulated PD-L1 expression showed improved responses to nivolumab (anti-PD-1) and pembrolizumab (anti-PD-L1) compared with patients with undetectable or low expression of PD-L1 (4, 10, 11). Echinoderm microtubule-associated protein-like 4–anaplastic lymphoma kinase (EML4-ALK) fusion is a key trigger in the upregulation of PD-L1 (12). No study reported the relationship of ROS1 fusion with PD-L1 expression despite its high similarity with ALK.

Currently, the pembrolizumab (anti-PD-1) is approved for use as first- and second- line therapy in patients with advanced NSCLC whose tumors express PD-L1 in immunohistochemistry analysis (10, 13). Nivolumab (anti-PD-1) and atezolizumab (anti-PD-L1) are both indicated for use as second-line therapies (10, 14). Durvalumab (anti-PD-L1) is approved as a maintenance therapy in patients with unresectable, stage 3 NSCLC whose disease has not progressed following concurrent platinum-based chemoradiotherapy (15). However, many issues are still not resolved regarding the biomarker status, choice in the first-line setting, immunotherapy in oncogene-addicted tumors, and how to combine immunotherapy with other agents.

The mechanism of the regulation of PD-L1 expression in ROS1 fusion primary tumor and acquired resistance to crizotinib is still unknown. Although many options are available for molecular targeted therapies of primary sensitive or secondary mutation of ROS1 tyrosine kinase domain drug resistance mutation, it remains unclear about the clinical potential of anti-PD-L1/PD-1 ICI therapy on ROS1 fusion and ROS1-G2032R crizotinib resistant mutation. Our aims of this study were 1) to explore the relationship between ROS1 fusion, ROS1 G2032R crizotinib resistant mutation and PD-L1, and 2) the clinical potential of anti-PD-L1 ICI therapy in ROS1-driven and drug-resistant ROS1 fusion G2032R mutation cell lines and mouse models. In this research, we share our insights related to ICI therapy by examining the role of ROS1 fusion and ROS1 G2032R resistance mutation on PD-L1 expression in NSCLCs by *in vitro* and *in vivo* experiments.

## Materials and Methods

### Cell Culture and Reagents

HCC78 (Cat NO.: CBP60100) and IL-3 producing WEHI-3 (Cat NO.: CBP60532) cell lines were obtained from Cobioer Company (http://www.cobioer.com/). The cell lines were cultured in Roswell Park Memorial Institute (RPMI) 1640 medium (Biological Industries) with 10% fetal bovine serum (FBS). Crizotinib (Cat NO.: S1086), Lorlatinib (Cat NO.: S7536), TPX-0005 (Cat NO.: S8583) and SHP099 (Cat NO.: S8278) purchased from Selleckchem. The HCC78 cell line was maintained in RPMI-1640 medium that contained Crizotinib at a starting concentration of 100 nM until a ﬁnal concentration of 2 mM over 10 months. Then, the resulting resistant cells were maintained in RPMI-1640 medium with 0.5 mM crizotinib and were designated as HCC78CR1, -2, -3, -4, and -5 cell lines. HCC78 and HCC78CR1-5 cell lines were authenticated by detecting the SLC34A2–ROS1 fusion using next-generation RNA sequencing. All cells were maintained under a humidified atmosphere of 5% CO_2_ at 37°C. For *in vitro* studies, crizotinib was dissolved in dimethyl sulfoxide (Sigma Aldrich) at 10 mM and stored at -80°C. Ba/F3, Ba/F3 ROS1 fusion harboring SLC34A2-ROS1 (Cat NO.: CBP73191) and Ba/F3 ROS1-G2032R harboring both ROS1 fusion and G2032R mutation (Cat NO.: CBP73192) were also obtained from Cobier Company. The Ba/F3 ROS1 fusion, Ba/F3 ROS1-G2032R harboring SLC34A2-ROS1 with G2032R crizotinib resistant mutation and IL-3 producing WEHI-3 cell lines were cultured in RPMI 1640 medium (Biological Industries) with 10% FBS. The IL-3-dependent mouse pro-B cell line, Ba/F3, was cultured in 10% FBS and 10% WEHI-3-conditioned medium as a source of IL-3. To obtain the WEHI-3-conditioned medium, WEHI-3 cells were seeded into culture medium (10^7^/100 mL) in the T75 culture flask and cultured for about 4 days until the color of medium turned almost yellow. The IL-3 containing medium was harvested, centrifuged, filtered with a microfilter system (0.2 μm), and kept frozen at –80°C.

### RNA Sequencing

Libraries for transcriptome sequencing (RNA sequencing) were constructed with NEB Next Ultra RNA Library Prep Kit for Illumina (NEB). Poly(A) tailed mRNA molecules were enriched from 1 μg total RNA with NEB Next Poly(A) mRNA Magnetic Isolation Module (NEB) kit. The mRNA was fragmented into approximately 200 base pair pieces. The first-strand cDNA was synthesized from the mRNA fragments reverse transcriptase and random hexamer primers, and then the second-strand cDNA was synthesized using DNA polymerase I and RNase H. The end of the cDNA fragment was subjected to an end repair process that included the addition of a single ‘A’ base, followed by ligation of the adapters. Products were purified and enriched by polymerase chain reaction (PCR) to amplify the library DNA. The final libraries were quantified using KAPA Library Quantification kit (KAPA Biosystems, South Africa) and an Agilent 2100 Bioanalyzer. After quantitative reverse transcription-polymerase chain reaction (RT-qPCR) validation, libraries were subjected to paired-end sequencing with pair end 150-base pair reading length on an Illumina HiSeq sequencer (Illumina). First, the sequenced reads in raw fastq format data were mapped to the hg19 *Homo sapiens* genome and transcriptome (gencode v19) using RNA STAR software (v2.4.0). The RNAseq datasets presented in this study can be found in online repositories. The names in the National Omics Data Encyclopedia and Project ID OEP000975 can be reached.

### RNA Interference

The HCC78 cell lines were plated at 40% to 50% confluence in 12-well plates and incubated for 24 h before transient transfection for 72 h with siRNAs mixed with Lipofectamine reagent (Invitrogen). The siRNAs specific for ROS1 mRNA (ROS1-1, 5′-ACACCCAAAUUAAUACCAA-3′; ROS1-2, 5′-UCAGCAAAUUCAACCACCA-3′) and a nonspecific siRNA (5′-GUUGAGAGAUAUUAGAGUU-3′) were obtained from RUIBIO Inc.

### Flow Cytometric Analysis

Cells were stained with biotinylated monoclonal antibodies against human PD-L1 (Biolegend) and with phycoerythrin-labeled streptavidin (eBiosciences) for flow cytometric analysis with Cytoflex instrument (Beckman coulter). The data were analyzed using FlowJo software.

### Western Blotting Analysis

Western blotting analysis was performed as most described. Protein estimation was performed by using Thermo Pierce™ BCA Protein Assay Kit (cat NO.: 23225). Rabbit polyclonal antibodies against human phosphorylated ROS1 (Y1604), phosphorylated EGFR (Y1068), phosphorylated ERK, phosphorylated signal transducer and activator of transcription 3 (STAT3), and glyceraldehyde-3-phosphate dehydrogenase were obtained from Cell Signaling Technology. All antibodies were used at a 1:1000 dilution. Horseradish peroxidase-conjugated goat antibodies against rabbit immunoglobulin G were obtained from Abcam. Immune complexes were detected with the use of Pierce Western Blotting Substrate Plus (Thermo Scientific) and Las-mini 4000 system (GE Inc.).

Ba/F3 Tumor Model: Ba/F3 ROS1 fusion and Ba/F3 ROS1-G2032R resistant mutation cell lines were implanted subcutaneously into the right flank of female C3H mouse (100 µl 107 cells in cell suspension in phosphate-buffered saline (PBS) buffer). Mice were treated once daily by oral gavage with vehicle (PBS buffer) or crizotinib (25 mg/kg) was administered orally to tumor-bearing mice daily for 28 consecutive days, and tumor volumes were monitored every 2 days for 4 weeks. L×W^2^×0.5 was used to calculate tumor volume (mm^3^). For the analysis of the effect of ICI therapy on ROS1 fusion or ROS1 G2032R resistance mutation, tumor-bearing animals were treated with a single dose of 100 µg per mouse by intratumoral injection.

## Results

### PD-L1 Expression Is Induced by ROS1 Fusion

**Figure 1:**
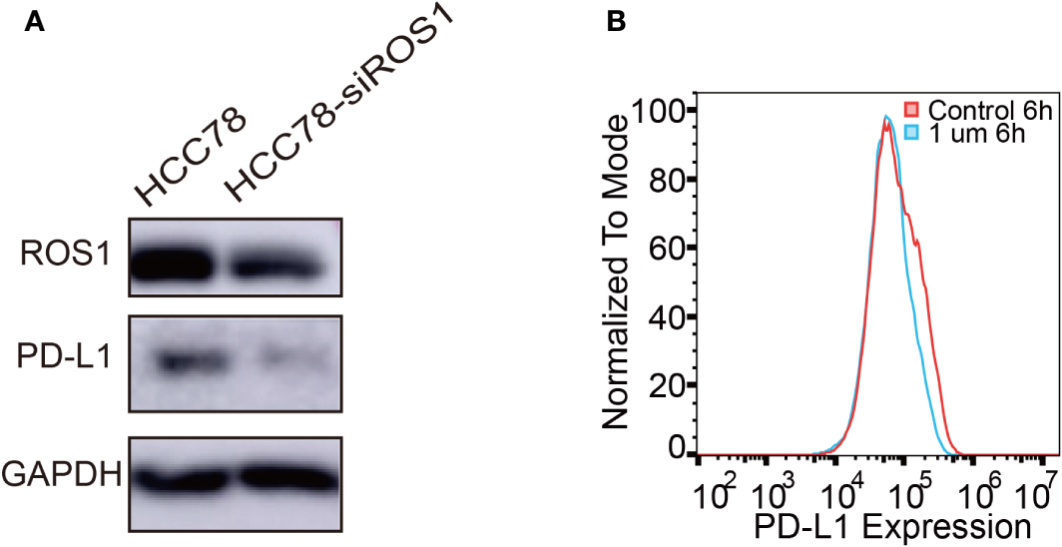
Programmed death-ligand 1 (PD-L1) expression induced by ROS1 fusion. **(A)** Western blot analysis of PD-L1 expression after specific interference of ROS proto-oncogene 1, receptor tyrosine kinase (ROS1) fusion; **(B)** Flow cytometry analysis of PD-L1 expression on the surface of HCC78 cells after 6 h of treatment with crizotinib at 1 µM.

In this study, HCC78 cell line was selected for its SLC34A2–ROS1 fusion gene, whereas the other lung cancer cell lines are wild type for ROS1. To investigate the effect of ROS1 fusion on PD-L1 expression, we conducted two cell experiments by transfecting HCC78 cells with siRNA specific for SLC34A2–ROS1 fusion or treating HCC78 cells with crizotinib. Immunoblot analysis with antibodies of the total forms of ROS1 confirmed the downregulation of ROS1 and PD-L1 expression after using siRNA specific for SLC34A2–ROS1 (**Figure 1A**). Flow cytometric analysis showed low PD-L1 expression on HCC78 cell surface treated by crizotinib (**Figure 1B**). Altogether, these results show that the expression of PD-L1 is closely associated with ROS1 fusion and its phosphorylation.

### Expression of PD-L1 in Crizotinib-Sensitive and Resistant HCC78 Cell Line

**Figure 2:**
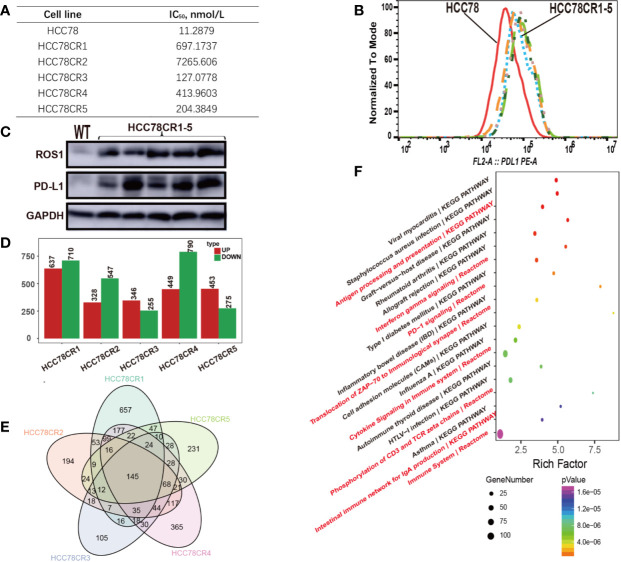
Crizotinib-resistant cells showing high ROS proto-oncogene 1, receptor tyrosine kinase (ROS1) and programmed death-ligand 1 (PD-L1) expressions. **(A)** Analysis of HCC78 and HCC78CR1–5 cell resistance characteristics by CCK8; **(B)** Flow cytometric analysis of PD-L1 surface expression in HCC78 and HCC78CR1–5 cells; **(C)** Western blot analysis of protein expression of ROS1 and PD-L1 in HCC78 and HCC78CR1–5 cells; **(D)** The number of upregulated and downregulated genes in HCC78CR1–5 compared with HCC78; **(E)** Venn plot analysis of HCC78CR1–5 differential genes; **(F)** Signal net of HCC78CR1–5.

To further explore the relationship of PD-L1 expression and ROS1 fusion in crizotinib resistance, we cultured five HCC78 crizotinib resistant cell lines (HCC78CR1–5). Crizotinib resistance of HCC78CR1–5 was detected by Cell Counting Kit-8 with half maximal inhibitory concentration (CCK8 IC50) (**Figure 2A** **)**. PD-L1 expression in HCC78 and HCC78CR1–5 was analyzed by flow cytometry (**Figure 2B** **)** and immunoblotting (**Figure 2C** **)** to confirm the cell surface and total expression, respectively. The HCC78CR1–5 sublines showed remarkably higher levels of PD-L1 than their parental HCC78 cell line. HCC78CR1–5 cell lines showed higher levels of PD-L1 protein compared with the HCC78 cell line, as shown by Western blotting, along with the upregulated expression of ROS1 fusion. These data overall suggest that the increased expression of PD-L1 is associated with ROS1 fusion.

To depict the complex relationship, we conducted the RNA sequencing experiment on HCC78 and HCC78CR1–5 sublines. The fusion and mutation status of ROS1 in HCC78 and HCC78CR1–5 cell lines were then assessed by RNA sequencing data. Although HCC78 harbors the ROS1 fusion, the HCC78CR1–5 sublines in our study lacked secondary drug resistance mutation. Every cell line harbored its own differential expression genes (**Figure 2D**), whereas 145 genes were shared by HCC78CR1–5 as indicated by Venn plot analysis (**Figure 2E** **)**. HCC78CR1 harbored the most genes, of which 234 were upregulated, and 466 were downregulated. HCC78CR1–5 shared 145 genes, which were speculated to be related to the expression of PD-L1 in acquired drug resistance without resistant mutation. Enrichment analysis of the pathway showed that the HCC78CR1–5 cell lines are more immune sensitive to PD-1 signaling, cytokine signaling in immune system, and immune system compared with the HCC78 cells (**Figure 2F**).

### ROS1 Fusion Induces Increased PD-L1 Expression Than ROS1 G2032R Mutation

**Figure 3:**
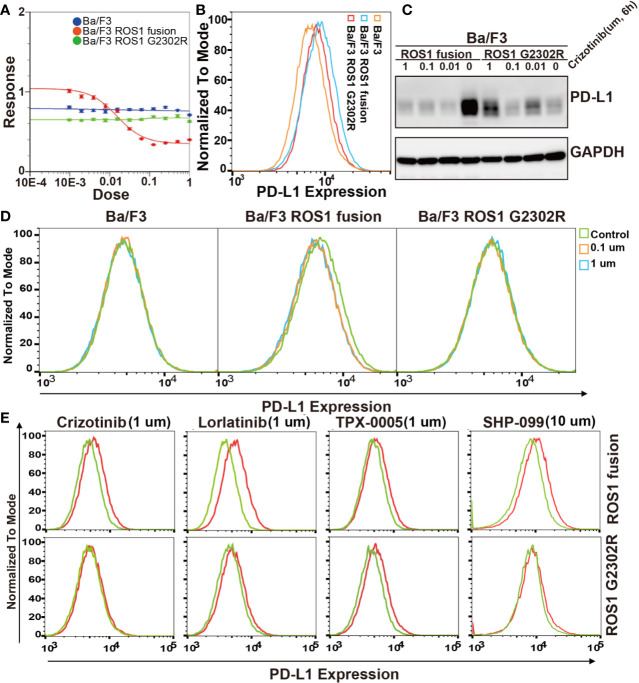
Effect of ROS proto-oncogene 1, receptor tyrosine kinase (ROS1) fusion and G2032R mutation resistance on programmed death-ligand 1 (PD-L1) expression. **(A)** Analysis of Ba/F3, Ba/F3 ROS1 fusion and Ba/F3 ROS1 G2032R crizotinib resistant characteristics by CCK8; **(B)** Flow cytometric analysis of PD-L1 surface expression in Ba/F3, ROS1 fusion, and ROS1 G2032R mutation resistance cell lines; **(C)** Flow cytometric analysis of PD-L1 surface expression in Ba/F3, ROS1 fusion, and ROS1 G2032R mutation resistant cell lines; **(D)** The protein expression of PD-L1 in ROS1 fusion and ROS1 G2032R mutation resistant cell lines after treatment with crizotinib; **(E)** PD-L1 expression after ROS1 inhibition by crizotinib, Lorlatinib, and TPX-0005 at 1 μM for 6 h or SHP2 (10 μM) by SHP099 for 10 h. Red line represents control group and Green line represents treatment group.

The ROS1 secondary resistance mutation, ROS1 G2032R, is associated with acquired crizotinib resistance in ROS1 fusion NSCLCs. To investigate the effect of ROS1 fusion and acquired drug resistance ROS1 G2032R mutation on PD-L1 expression, Ba/F3 ROS1 fusion and Ba/F3 ROS1 G2032R resistance mutation stable cell line were constructed. Crizotinib resistance in Ba/F3, Ba/F3 ROS1 fusion and Ba/F3 ROS1 G2032R resistance mutation cell was detected by CCK8 IC50 (**Figure 3A**). Flow cytometric analysis results revealed that ROS1 fusion also increased the level of PD-L1 expression at the surface of Ba/F3 ROS1 fusion, but a lower level was noted in ROS1 G2032R mutation (**Figure 3B**). To further explore this effect, we conducted flow cytometric analysis on the PD-L1 expression after treatment using 0, 0.1, and 1 µm crizotinib for 6 h. The PD-L1 expression was lower in Ba/F3 ROS1 fusion than the control group in **Figure 3C**, whereas no changes were observed in Ba/F3 and Ba/F3-ROS G2032R in **Figure 3D**.

We further confirmed this result in Ba/F3 ROS1 fusion and Ba/F3 ROS1 G2032R mutation. To exclude the possibility that these results were due to nonspecific effects of crizotinib, we further investigated the effect of the phosphorylation of ROS1 and SHP2 on PD-L1 expression in Ba/F3 ROS1 fusion and Ba/F3 ROS1 G2032R cells by crizotinib, TPX-0005 and Lorlatinib at 1 um for ROS1 and SHP099 at 10 um for SHP2 (**Figure 3E**) after 6 h. The inhibition of the phosphorylation of SHP2 resulted in a decrease in the abundance of PD-L1 surface expression in Ba/F3 ROS1 fusion cells. Altogether, these findings indicate that PD-L1 expression increased because of the increased ROS1 tyrosine kinase activity and SHP2 phosphorylation.

### ROS1–SHP2 Pathway Regulates PD-L1 Expression

**Figure 4:**
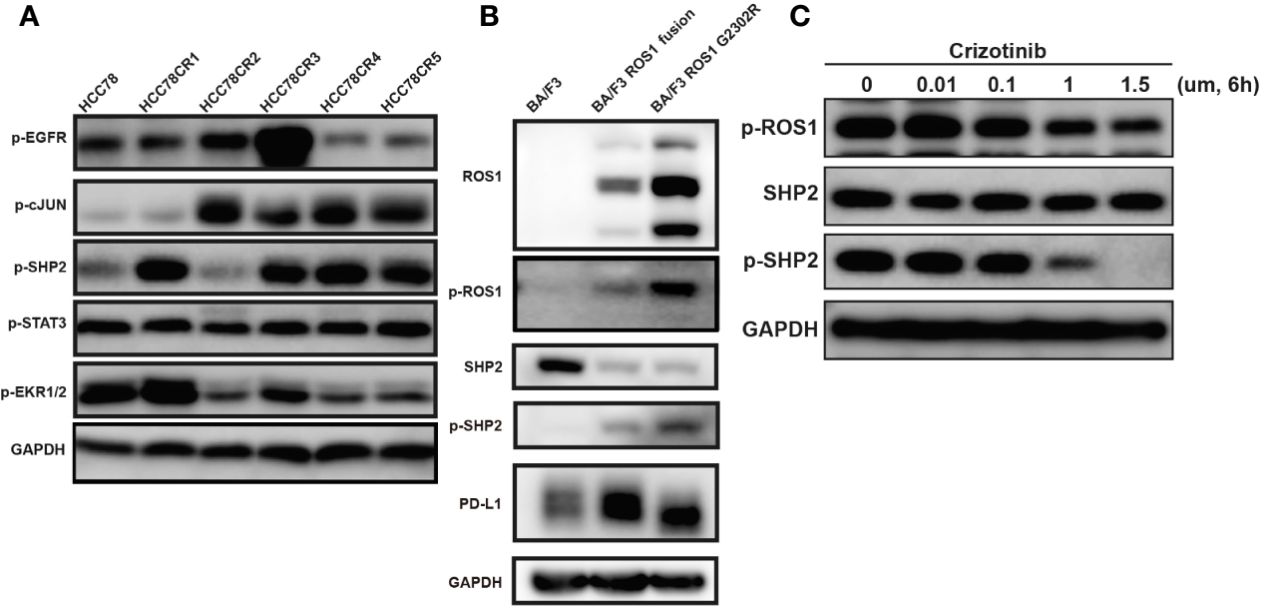
Phosphorylation of epidermal growth factor receptor (EGFR), c-JUN, SHP2, STAT3 and Erk1/2. **(A)** Phosphorylation of EGFR, c-JUN, SHP2, STAT3, and Erk1/2 in HCC78CR1–5 cells compared with HCC78 cell line; **(B)** phosphorylation of ROS proto-oncogene 1, receptor tyrosine kinase (ROS1) and SHP2 with programmed death-ligand 1 (PD-L1) expression in Ba/F3, ROS1 fusion, and ROS1 G2032R mutation resistance cell lines; **(C)** The effect of crizotinib on SHP2 and ROS1 phosphorylation.

To identify the signaling pathways and further validation the role of ROS1-SHP2 pathway in the PD-L1 expression regulation, we detected the expression of SHP2, c-Jun, ERK1/2, and EGFR after the examination of published papers involving HCC78, crizotinib, and the regulation mechanism of PD-L1 expression (16). In HCC78CR1–5, the phosphorylations of SHP2 and the downstream targets of c-Jun were higher than those of the HCC78 cell line (**Figure 4A**). We further confirmed this result in Ba/F3, Ba/F3 ROS fusion, and Ba/F3 ROS G2032R (**Figure 4B**). As mentioned, we examined the effect of crizotinib on endogenous PD-L1 expression in the HCC78 cell line. Crizotinib blocked the phosphorylation of ROS1 and SHP2 in Ba/F3 ROS1 fusion cells (**Figure 4C**), and this effect was accompanied by the downregulated amounts of PD-L1 surface protein. Altogether, these findings indicated that PD-L1 expression increased because of the increased ROS1 tyrosine kinase activity and SHP2 phosphorylation.

### Ba/F3 ROS1 Fusion-Bearing Mouse Shows Stronger Immunogenicity in Immune Micro-Environment Than Ba/F3 ROS1 G2032R

**Figure 5:**
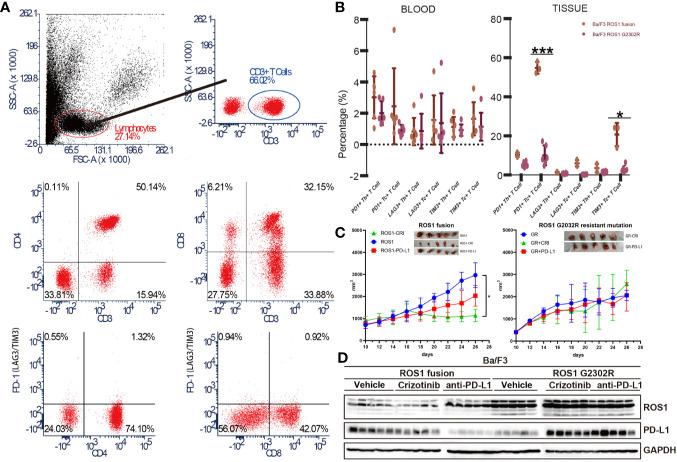
Ba/F3 ROS1 fusion bearing mouse showed an increased response to ant- programmed death-ligand 1 (PD-L1) immune checkpoint inhibitor (ICI) therapy than Ba/F3 ROS1 G2032R resistant mutation. **(A)** Flow cytometry and analysis strategies used in this study; **(B)** Flow cytometric analysis of PD-1, lymphocyte activation gene 3 (LAG-3) and T-cell immunoglobulin mucin-3 (TIM3) in Ba/F3 in ROS proto-oncogene 1, receptor tyrosine kinase (ROS1) fusion and Ba/F3 ROS1 G2032R mutation of blood and tissue samples based on T cell type; **(C)** Tumor size changes in Ba/F3 ROS1 fusion in Ba/F3 ROS1 G2032R mutation C3H mice (n = 5) after crizotinib or anti-PD-L1 therapy. ROS1+CRI: Ba/F3 ROS1 fusion+crizotinib; ROS1: Ba/F3 ROS1 fusion; ROS1+PD-L1: Ba/F3 ROS1 fusion+anti-PD-L1; GR: Ba/F3 G2032R mutation; GR+CRI: Ba/F3 G2032R mutation+crizotinib; GR+PD-L1: Ba/F3 G2032R mutation+ anti-PD-L1; **(D)** Western blotting analysis of ROS1 and PD-L1 in Ba/F3 ROS1 fusion and Ba/F3 ROS1 G2032R mutation of tissue samples after crizotinib and anti-PD-L1 ICI therapy. p < 0.05 was considered statistically significant and denoted as follows: *p < 0.05, ***p < 0.001.

Immune micro-environment is a key component of TME, especially in the context of ICI therapy. To explore the effect of the upregulation of PD-L1 between Ba/F3 ROS1 fusion and ROS1 G2032R resistant mutation on immune micro-environment, we constructed Ba/F3 ROS1 and ROS1 G2032R bearing C3H mice with complete immune capacity. Flow cytometric analysis of common immune checkpoint PD-1, lymphocyte activation gene 3 (LAG-3), and T-cell immunoglobulin mucin-3 (TIM3) on Th and Tc cells was performed in blood and tissue (**Figure 5A**). A significant difference was found in PD-1, LAG-3 and TIM3 on T cells of tissue samples between Ba/F3 ROS1 fusion and ROS1 G2032R bearing C3H mice, and the same trend was observed in blood samples with PD-1, LAG-3 and TIM3 (**Figure 5B**). The ROS1 fusion group showed tumor shrinkage after treatment with crizotinib and anti-PD-L1 but not the G2032R group (**Figure 5C** **)**. Western blotting analysis showed that after the ROS1 fusion group was treated with crizotinib and anti-PD-L1, the expression of PD-L1 in the tumor immune micro-environment decreased, whereas the expression was up-regulated in the G2032R group (**Figure 5D**).

## Discussion

Given its convenient detection of targets and high screening rate among beneficial population, molecular targeted therapy plays an important role in the treatment of NSCLCs. Despite the lack of suitable biomarkers, accumulating evidence suggests that PD-L1 is a good indicator for anti-PD-L1/PD-1 ICI therapy. In general, the expression of PD-L1 in lung cancer tissue is still an effective predictor for the anti-PD-L1 checkpoint inhibitor therapy. For PD-L1 > 50%, single-agent pembrolizumab (pembro) is the standard (17); combination of chemotherapy (chemo)/pembro for patients who needs rapid response; nivo/ipi for high TMB is discouraged unless comparative data are available. For PD-L1 between 1%–49%, the combination of chemo/pembro is pending for approval; chemo/bev/atezo is an option; nivo/ipi combination for patient with high TMB is unsuitable for chemotherapy. For 0% PD-L1, chemo may still be the standard; TMB testing is the option for this group for the selection of patients with nivo/ipi or chemo/nivo combination treatment (18). Our study showed that the treatment by crizotinib of HCC78 cells resulted in the downregulated PD-L1 surface expression at the protein level. Conversely, the specific ROS1 RNA interference suppressed the expression of PD-L1 in HCC78 cell line positive for SLC34A2–ROS1 fusion. Our results thus indicate that PD-L1 expression is induced in NSCLC cells by the ROS1 fusion, with this induction being a key event in the pathogenesis of ROS1 fusion NSCLCs. Our study further showed the upregulation of PD-L1 in HCC78 crizotinib resistant cell lines (HCC78CR1–5) with none drug resistant mutation on ROS1 fusion. Our study further confirmed a recent study (19) by Zheng et al. published on 30 March 2020, which shows that ROS1 fusion primary NSCLC tumor was significantly associated with the up-regulation of PD-L1 expression.

Expression of PD-L1 is induced *via* oncogenic signaling pathways, such as RAS/RAF/MEK/mitogen-activated protein kinase-ERK (20–23), phosphatidylinositol-3 kinase/phosphatase and tensin homolog/Akt/mammalian target of rapamycin (24–28), EGFR (8, 29–31), and EML4-ALK pathways (23, 32), in many cancer types. In our study, the expression of PD-L1 depended on the ROS1 fusion along with the over-phosphorylation of SHP2 in ROS1 fusion or ROS1 G2032R resistant mutation Ba/F3, and was higher in ROS1 fusion than the ROS1 G2032R resistant mutation. Given that the ROS1 tyrosine kinase activates the SHP2 signaling pathway (33), we focused on the possible role of the SHP2 pathway and its downstream signaling pathways in the induction of PD-L1 expression in ROS1 fusion NSCLCs. The results show that SHP2 and c-Jun pathways regulate PD-L1 expression in ROS1 fusion crizotinib resistant lung cancer cells, indicating that distinct oncogene ROS1 possesses signaling pathways for the regulation of PD-L1 expression in NSCLCs. A immunohistochemical analysis showed that PD-L1 expression was higher in NSCLC tumor specimens positive for ALK rearrangement than in those negative for ALK translocation (23). Given that PD-L1 expression in tumors is correlated with the likelihood of a response to therapies that target the PD-1–PD-L1 interaction, further studies are warranted to evaluate the efficacy of immunotherapies, such as treatment with ICI therapy, for such oncogene-driven NSCLCs. PD-1 has an important role in regulating T cell responses and have proven to be effective targets in NSCLCs where constitutive co-inhibitory receptor expression on T cells dampens effector T cell responses. Unfortunately, many patients still fail to respond to anti-PD-1 therapy. Lag-3 and Tim-3 being explored in clinical trials were the next wave of co-inhibitory receptor targets. Increased understanding of the specialized functions of these receptors after different oncogenic background will inform the rational application of therapies that target these receptors to the clinic.

Although, it has been designed carefully, our study has some limitations which can be listed as follows. Firstly, we focus on the change of TME on crizotinib of PD-L1 expression or on crizotinib resistance not at the combination of crizotinib with anti-PD-L1 ICI therapy. Secondly, we conduct the study on ROS1 fusion type NSCLCs, whether it can be referred to other fusion type or molecularly target is unknown. Thirdly, we demonstrated the distribution of exhausted T cells in subcutaneous xenograft mouse models. This subcutaneous tumor model is lack of vessel formation and underestimating the impact of the lung environment. Additionally, Ba/F3 based ROS1 fusion cells might have a different transcriptional background compared with pulmonary epithelial cells. Therefore, further pulmonary metastasis tumor models are needed in the future further study. A clinical treatment line for advanced ROS1-rearranged lung cancer was summarized by Alice T. Shaw et al (34). Our study gives a new insights of ICI therapy of this line mainly of ROS1 fusion and G2032R NSCLCs, so we choose Ba/F3 cell lines as the control group. The crizotinib was still the only first-line choice for ROS1 fusion type NSCLCs. Our study focused on the tumor microenvironment changes and its clinical potential for anti-PD-L1 therapy on ROS1 fusion NSCLCs. The results show that ROS1 fusion NSCLCs may have a good clinical response to anti-PD-L1 therapy because of the upregulation of PD-L1 and the increase of CD3+PD-1+ T cells, ROS1 G2032R resistant mutation NSCLCs may not benefit from anti-PD-L1 therapy. Based on our findings, anti-PD-1/PD-L1 ICI treatments should be applied as the first line therapy for NSCLC patients with ROS1 fusion while not in ROS1 G2032R mutation type NSCLCs. In our study, HCC78CR1-5 cell lines did not harbor the secondary resistant mutation detected by RNA-sequencing which indicate a non-mutation acquire crizotinib resistance mechanisms. And based our findings, anti-PD-1/PD-L1 ICI immunotherapy would be a more effective treatment modality for this type NSCLCs patients.

In conclusion, by preclinical experiment using SLC34A2–ROS1 fusion HCC78 and crizotinib-resistant cell lines, we have identified ROS1-positive fusion as a key determinant of PD-L1 expression in NSCLCs after crizotinib acquired resistant mutation G2032R. We further showed that ROS1 modulates PD-L1 expression *via* common downstream pathways mediated by the ROS1–SHP2 pathway. Our findings indicate that oncogenic drivers play a direct role in the induction of PD-L1 expression and thereby contribute to immune escape of NSCLCs. Our results also indicate that ROS1 fusion NSCLCs show a beneficial ICI therapy response, which lessens in ROS1 G2032R resistance mutation, thus implying that driver genes should be considered when conducting anti-PD-L1/PD-1 ICI therapy to manage lung cancer treatment.

## Data Availability Statement

Data Access: All the RNA-sequencing data of HCC78 and HCC78CR1-5 have been deposited at the National Omics Data Encyclopedia (NODE, https://www.biosino.org/node/index), which is hosted by the Chinese Academy of Sciences (CAS), under the Project ID OEP000975.

## Ethics Statements

The animal study was reviewed and approved by the ethics committee of the Chinese Academy of Medical Sciences and Peking Union Medical College. 

## Author Contributions

LC, JD, and ZW participated in the design of the study, performed experiments and participated in the writing of the paper. LQ, SL, CW, and JZ participated in the design of the study and performed experiments. JW and HB designed the study and participated in the writing of the paper. All authors contributed to the article and approved the submitted version.

## Funding

This work was supported by the National Key Research and Development Project (2019YFC1315700), the National Natural Sciences Foundation Key Program (81630071), the National Natural Science Foundation of China (81771689 and 81900194), the Ministry of Education Innovation Team Development Project (IRT-17R10), the CAMS Key Lab of Translational Research on Lung Cancer (2018PT31035), the CAMS Innovation Fund for Medical Sciences (CIFMS 2016-I2M-3-008), the Aiyou Foundation(KY201701), and the Wujieping Foundation(320675018477).

## Conflict of Interest

The authors declare that the research was conducted in the absence of any commercial or financial relationships that could be construed as a potential conflict of interest.
